# The Effect of a Care Bundle on Aggression Management in Patients with Psychotic Disorders: A Randomized Controlled Trial

**DOI:** 10.1192/j.eurpsy.2026.10628

**Published:** 2026-07-31

**Authors:** M. Bulut, N. Yıldırım

**Affiliations:** Department of Psychiatric and Mental Health Nursing, Bolu Abant Izzet Baysal University, Bolu, Türkiye

## Abstract

**Introduction:**

There is a clear gap between international guidelines, which recommend replacing coercive aggression management methods with alternative interventions, and their inconsistent and unsystematic implementation in clinical practice. This situation underscores the need for evidence-based aggression management strategies. The care bundle approach, designed to standardize care and improve outcomes, is a promising solution.

**Objectives:**

This study aimed to develop a care bundle for the management of aggression in patients diagnosed with schizophrenia and receiving inpatient treatment and to evaluate its clinical outcomes.

**Methods:**

The study was conducted in two phases. 1) the aggression management care bundle was developed by conducting a systematic literature review according to the Bundle Design Guidelines. 2) the six-element care bundle was implemented in a male psychiatric ward of a regional hospital using a sequential randomized controlled trial design, and the outcomes were evaluated (Figure 1). The study was conducted with 24 patients (12 in the experimental group and 12 in the control group). Data were collected using the Patient Information Form, Buss-Perry Aggression Scale, Brøset Violence Checklist, Overt Aggression Scale, and Care Bundle Compliance Follow-up Schedule. Data were analyzed using Pearson’s chi-square test, independent samples t-test, Mann-Whitney U test, paired samples t-test, two-way ANOVA for multifactor analyses, and linear mixed model analyses for mixed models.

**Results:**

Findings indicated a significant reduction in the experimental group’s total Buss-Perry Aggression Scale score and all subdimensions following care bundle implementation (p=0,001). The control group also exhibited decreases in total aggression (p=0,007), physical aggression (p=0,001), and anger scores (p=0,001). Intergroup pre-test–post-test differences were statistically significant in favor of the experimental group (p<0,001). Although Brøset Violence Checklist scores declined over time in both groups, no significant intergroup difference was observed (p>0,05). It was found that the BVC total score, irritability, boisterous, physical threat, and attack on objects sub-dimensions were affected by daily/momentary changes, while the confusion and verbal threat sub-dimensions were affected by the patient’s standard/permanent characteristics. According to the Overt Aggression Scale, aggressive incidents (control: 9, experimental: 6) and interventions applied did not differ significantly between groups (p>0,05). The care bundle demonstrated high clinical utility, with a 90,15% compliance rate.

**Image 1:**
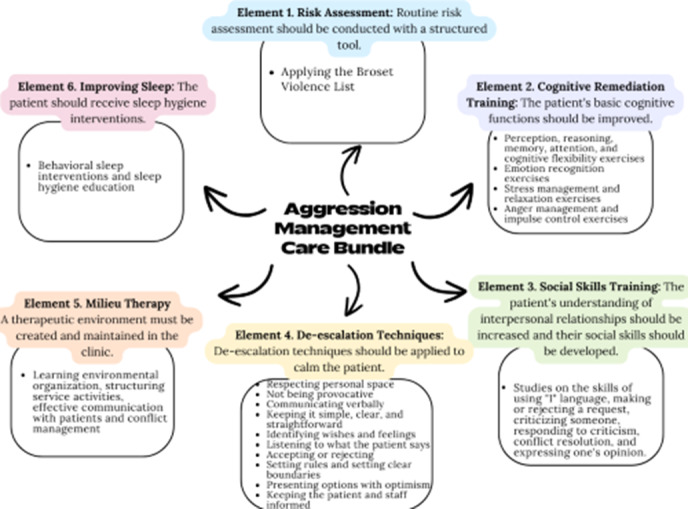
Image 1: Long description.

**Conclusions:**

The aggression management care bundle was found to be an effective and applicable for male patients with schizophrenia. It is recommended that the care bundle be applied to larger samples, individuals with different psychiatric diagnoses, and female patients.

This registration was supported by the Scientific and Technological Research Council of Turkey (TUBITAK) under the Grant Number 1919B022509487. The authors thank TUBITAK for their support.

**Disclosure of Interest:**

None Declared

## Long descriptions

### Image 1: Long description

At the center of the diagram is the text Aggression Management Care Bundle. Six curved arrows point outward to specific intervention elements.

* Element 1. Risk Assessment (Top): Routine risk assessment should be conducted with a structured tool. Action: Applying the Broset Violence List.

* Element 2. Cognitive Remediation Training (Top-Right): The patient's basic cognitive functions should be improved. Actions include perception, reasoning, memory, attention, and cognitive flexibility exercises; emotion recognition exercises; stress management and relaxation exercises; anger management and impulse control exercises.

* Element 3. Social Skills Training (Bottom-Right): The patient's understanding of interpersonal relationships should be increased and their social skills should be developed. Actions include studies on the skills of using I language, making or rejecting a request, criticizing someone, responding to criticism, conflict resolution, and expressing one's opinion.

* Element 4. De-escalation Techniques (Bottom): De-escalation techniques should be applied to calm the patient. Actions include respecting personal space, not being provocative, communicating verbally, keeping it simple, clear, and straightforward, identifying wishes and feelings, listening to what the patient says, accepting or rejecting, setting rules and setting clear boundaries, presenting options with optimism, and keeping the patient and staff informed.

* Element 5. Milieu Therapy (Bottom-Left): A therapeutic environment must be created and maintained in the clinic. Actions include learning environmental organization, structuring service activities, effective communication with patients and conflict management.

* Element 6. Improving Sleep (Top-Left): The patient should receive sleep hygiene interventions. Actions include behavioral sleep interventions and sleep hygiene education.

Navigate back to Image 1:.

